# Difficult Closure of Open‐Window Thoracostomy After Drainage of an Infected Pulmonary Bulla

**DOI:** 10.1002/rcr2.70500

**Published:** 2026-02-02

**Authors:** Kazuki Sato, Taiki Sato, Masahiro Miyajima, Hirofumi Uehara

**Affiliations:** ^1^ Department of Thoracic Surgery Hakodate Goryoukaku Hospital Hakodate Japan; ^2^ Department of Thoracic Surgery Sapporo Medical University Sapporo Japan

**Keywords:** infected pulmonary bullae, open window thoracostomy, percutaneous drainage, thoracic surgery

## Abstract

Percutaneous drainage can be used to treat infected pulmonary bullae unresponsive to antibiotics; however, it carries a high risk of bronchopleural fistula and empyema. We report the case of an elderly man with an infected pulmonary bulla successfully treated with percutaneous drainage followed by open‐window thoracostomy (OWT) for persistent air leakage. Although the infection was controlled, closing the OWT was challenging due to recurrent bronchial fistulae despite repeated surgeries. This case highlights the need to carefully identify and close all bronchial openings in the cyst wall to achieve successful closure after OWT in patients with severely infected bullae.

## Graphical abstract

This report describes an elderly man with an infected pulmonary bulla successfully treated with percutaneous drainage followed by open‐window thoracostomy (OWT) for persistent air leakage. Despite infection control, repeated failures of window closure occurred due to newly appearing bronchial fistulae. The case highlights the importance of meticulous identification and closure of all bronchial communications to achieve definitive healing after OWT performed for an infected pulmonary bulla.

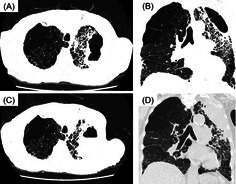

## 1 Introduction

Several reports have described percutaneous drainage for infected pulmonary bullae unresponsive to antibiotic therapy; however, complications such as bronchopleural fistula and empyema remain major concerns [1]. Complete control of the bronchopleural fistula is crucial for successful closure after open‐window thoracostomy (OWT) [2]. We report a case in which percutaneous drainage was performed as a life‐saving procedure for a severe infected pulmonary bulla; however, achieving closure after OWT for post‐drainage fistulous empyema was challenging.

## 2 Case Report

**FIGURE 1:**
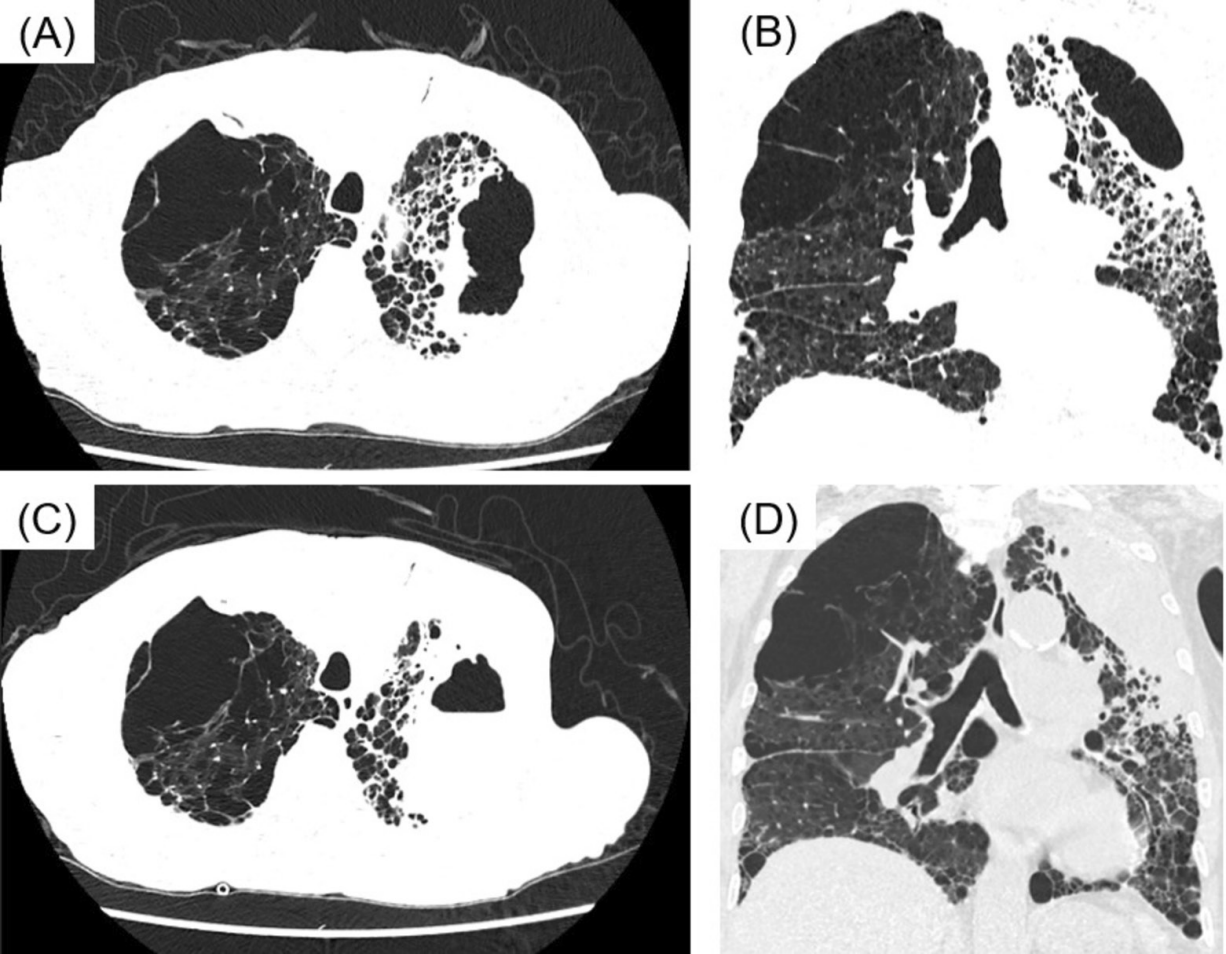
Chest computed tomography images. (A) Axial chest computed tomography image on admission. (B) Coronal chest computed tomography image on admission. (C) Axial chest computed tomography image on hospital Day 15. (D) Coronal chest computed tomography image on hospital Day 15.

The patient was a man in his 70s with a history of emphysema. He presented with fever and was admitted with pneumonia. Laboratory tests on admission revealed a positive Aspergillus antigen test; however, blood and sputum cultures, including those for acid‐fast bacilli, were negative. Chest computed tomography (CT) revealed an infiltrative shadow in the left upper lobe (Figure 1A,B). Although antibiotic therapy with piperacillin–tazobactam and antifungal therapy with micafungin were initiated after admission, his condition did not improve, and chest CT on hospital Day 15 revealed progression of the infiltrative shadow and fluid accumulation within the bulla in the left upper lobe, suggesting an infected pulmonary bulla (Figure 1C,D).

**FIGURE 2:**
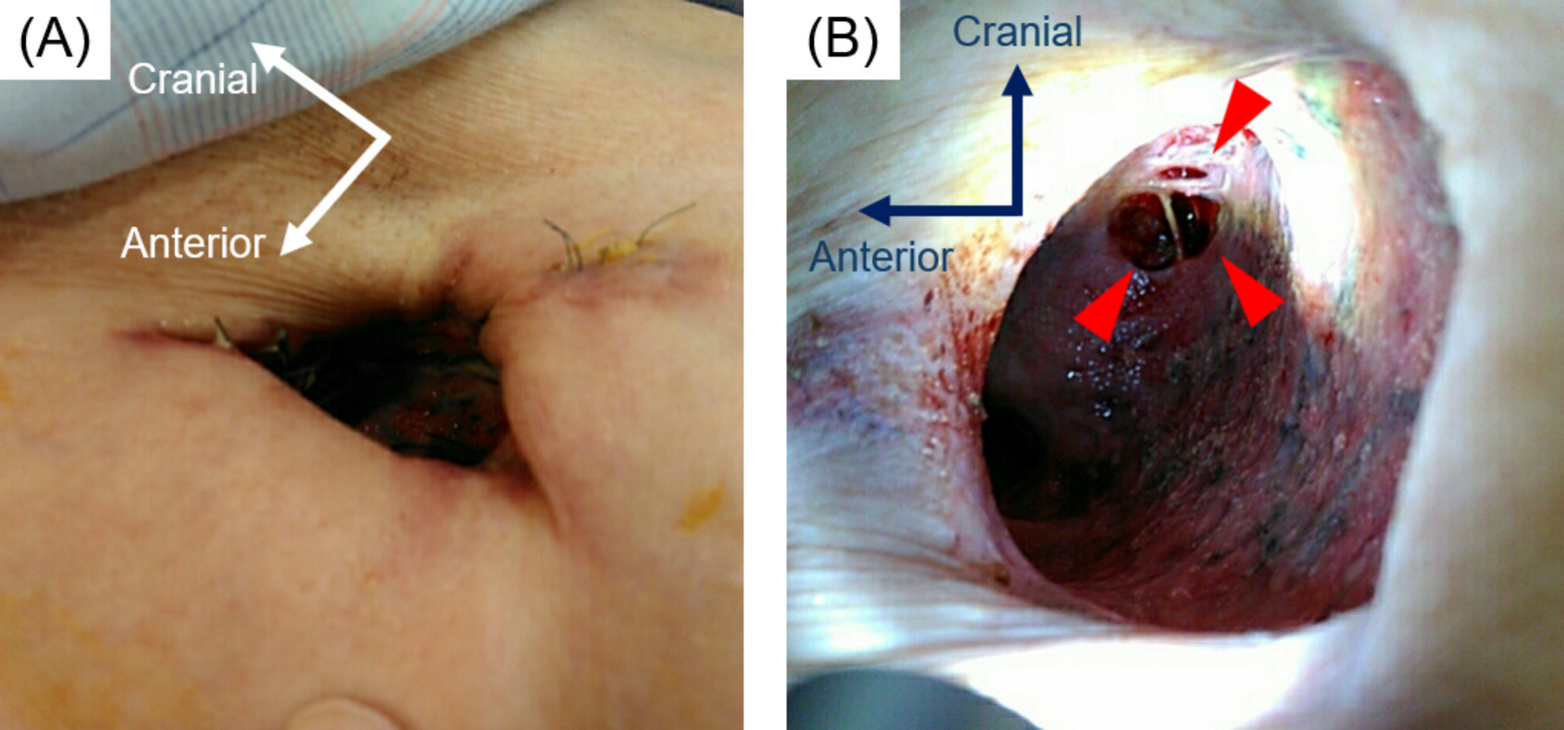
(A) Postoperative external view following open‐window thoracostomy. (B) Postoperative external view following open‐window thoracostomy. The red arrowheads indicate the fistulae at the base of the bulla.

**FIGURE 3:**
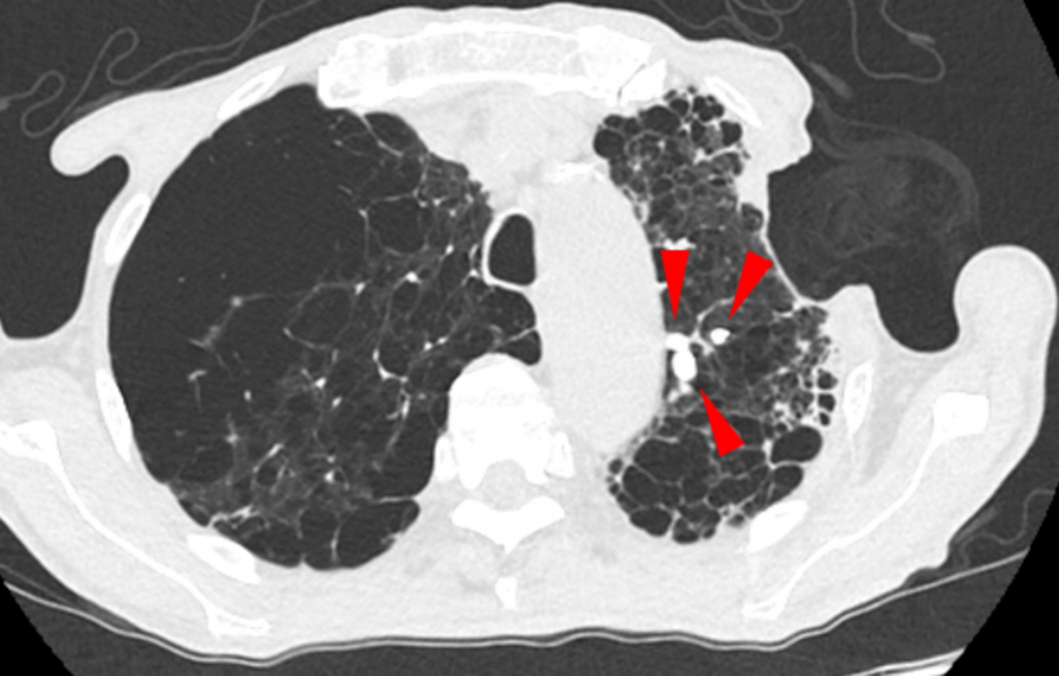
Computed tomography after endobronchial Watanabe spigot (EWS) placement. The red arrowheads indicate the EWS plugs.

CT‐guided percutaneous drainage of the infected bulla was performed on hospital Day 16 under local anaesthesia. The pneumonia and infected cavity improved; however, air leakage through the drainage catheter persisted. Therefore an OWT was performed on Day 30 to manage the continuous air leakage and control the infection. A skin incision was made in the axillary region over the left upper lobe, and the second and third ribs were resected to create a window (Figure 2A). The patient's postoperative course was uneventful, and he was discharged. As the infection was controlled, window closure was planned 6 months after surgery. Because air leakage from the fistula persisted (Figure 2B), endobronchial Watanabe spigot (EWS) placement was performed to reduce the leakage (Figure 3). Following EWS placement, the air leak was reduced; however, a small residual air leak remained. Subsequently, a pedicled latissimus dorsi flap was mobilised to cover the fistula and obliterate the dead space at the open‐window site, thereby achieving closure. At an outpatient visit 10 days after discharge, air leakage and fistula recurrence through the skin flap were observed; therefore, reoperation was performed. The previously packed latissimus dorsi muscle was dissected, and the base of the bulla was exposed. During the leak test, air leakage was detected at a site other than the previously closed bronchial fistula. After identifying the leakage site, the site was sutured, and a free fascia lata graft was harvested and sutured to the base of the bulla. The dissected latissimus dorsi muscle was repacked into the space, and the wound was closed. Although a small amount of air leak was noted postoperatively from the drain, it gradually improved. However, on postoperative Day 9, infection at the drain insertion site and air leakage recurred. *Pseudomonas aeruginosa* was isolated from the wound culture, suggesting a transbronchial infection. On postoperative Day 10, the wound was incised for drainage and the patient was discharged with the wound left open. At present, the drainage is satisfactory, and the patient's course is stable without worsening the infection; therefore, closure has not been performed, and the patient is being managed with careful follow‐up.

## 3 Discussion

In this case, percutaneous drainage served as a life‐saving intervention for a severely infected pulmonary bulla that was unresponsive to antibiotic therapy. Although infection control was achieved, the subsequent development of a fistulous empyema complicated the clinical course and made definitive closure difficult.

We successfully saved the life of a patient with a severely infected pulmonary bulla unresponsive to antibiotic therapy by performing percutaneous drainage. Currently, there is no clear consensus regarding the optimal management of infected pulmonary bullae that are unresponsive to antibiotic therapy. While some reports have described the efficacy of percutaneous drainage [3], concerns remain regarding potential complications, including pneumothorax and bronchopleural fistula [1]. In cases such as the present case, multiple bronchioles are often connected to the cystic cavity, increasing the risk of developing fistulous empyema. However, in patients with systemic deterioration and progressive infections, percutaneous drainage under local anaesthesia may serve as an effective, life‐saving measure for severely infected pulmonary bullae. In the present case, air leakage occurred following drainage, leading to a fistulous empyema. An OWT was subsequently performed for infection control in the presence of a bronchopleural fistula. This procedure proved effective for managing fistulous empyema following percutaneous drainage.

In this case, closure could not be achieved because controlling the bronchial fistula after OWT was challenging. According to Naclerio and Langer, complete management of multiple bronchial openings in the cyst wall is crucial for preventing postoperative recurrence in cases of giant bullae [4]. Similarly, when multiple bronchioles communicate with the cystic cavity, complete closure of all bronchioles is essential to achieve successful closure. In the present case, although the visibly patent bronchioles were treated, a new bronchiole subsequently became patent, resulting in recurrent air leakage and failure of closure. However, once the initially responsible bronchus is closed and the intrapulmonary pressure increases, previously functionally occluded bronchioles may become patent. In our case, this secondary manifestation of latent bronchial fistulae was likely the main reason for the difficulty in achieving definitive closure. In summary, this case highlights the importance of meticulous identification and closure of multiple bronchial fistulae in the surgical management of infected giant pulmonary bullae.

In conclusion, although percutaneous drainage can be a vital life‐saving option for infected pulmonary bullae, the presence of multiple bronchial communications poses a major challenge to subsequent closure. Detailed intraoperative exploration and complete closure of all bronchial fistulae are indispensable to achieve definitive healing after OWT.

## Author Contributions

Kazuki Sato drafted the manuscript. Kazuki Sato, Taiki Sato and Hirofumi Uehara were involved in the clinical management of the patient. Hirofumi Uehara and Masahiro Miyajima were responsible for revising the manuscript critically for important intellectual content. All authors read and approved the final manuscript.

## Consent

The authors declare that written informed consent was obtained for the publication of this manuscript and accompanying images using the consent form provided by the Journal.

## Conflicts of Interest

The authors declare no conflicts of interest.

## Data Availability

Data sharing not applicable to this article as no datasets were generated or analyzed during the current study.
